# From male caduco to altered consciousness: two Medici recipes through the lens of modern consciousness science

**DOI:** 10.1007/s10072-026-09309-5

**Published:** 2026-08-07

**Authors:** Angela Comanducci

**Affiliations:** Fondazione Don Carlo Gnocchi ETS, Florence, Italy

## Abstract

This article examines two therapeutic recipes from the collection of Anna Maria Luisa de’ Medici (1667–1743), the last Medici princess: the *Polvere da Infantiglioli* against the falling sickness (*male caduco*) and a preparation of amber essence. Together, they provide a case study of how early modern medicine approached sudden, reversible episodes of impaired awareness and responsiveness, phenomena that modern neurology situates at the intersection of epileptology and consciousness science. Drawing on archival sources from the *Archivio di Stato di Firenze*, this paper examines the language, ingredients, and stated uses of these preparations and interprets them through the modern distinction between the level of consciousness and its content. This study argues that the two recipes reflect an undifferentiated therapeutic practice directed at functions that contemporary consciousness science would distinguish, and that the empirical observation and attempted reversal of transient losses of responsiveness long preceded any mechanistic account of such phenomena. Their circulation through Anna Maria Luisa’s courtly and epistolary networks helped sustain her political relevance during the dissolution of the Medici dynasty and highlights the role of aristocratic women in preserving and transmitting medical knowledge outside institutional medicine.

**Supplementary information:**

The online version contains supplementary material available at https://doi.org/10.1007/s10072-026-09309-5.

In July 1735, Baroness Massimiliana Moltke^1^ wrote from Vienna to Florence to thank Anna Maria Luisa de’ Medici^2^ (1667–1743), the last Medici princess, for a powder that had cured three noble children of violent convulsions (*Archivio di Stato di Firenze* [ASF], *Mediceo del Principato* 6341, fol. 126r). The physicians, the Baroness wrote, had already *“abandoned”* them; months later, the children remained in perfect health [1].

This brief epistolary exchange offers a starting point for a narrower question: what did early modern medicine actually do, in practice, when faced with the sudden, reversible episodes of impaired awareness and responsiveness, phenomena that modern neurology would describe as seizure-related alterations of consciousness? This article uses two archival recipes, the *Polvere da Infantiglioli* [*Infantiglioli* powder] against the falling sickness and a preparation of amber essence, as a case study in how pre-modern therapeutic practice approached clinical phenomena that today lie at the intersection of epileptology and consciousness science.

The remedy’s name is itself linguistically revealing: *“Ricetta per fare la Polvere da Infantiglioli contro il Male Caduco*,* o sia mitrito”* [Recipe for making the *Infantiglioli* powder against the *male caduco*, or *mitrito*] [1]. Three vernacular terms carry the clinical sense: *infantiglioli*, *mitrito and male caduco*.

*Infantiglioli* denotes, in Italian usage, “*the popular name for infantile eclampsia and*,* more generally*,* for any type of infantile convulsive attack*” (*Devoto-Oli*, *Il Dizionario della Lingua Italiana*). 

*Mitrito* (or *metrito*) is a regional term equivalent to epilepsy or an epileptic crisis; it also denotes convulsive crises more generally, with particular reference to those provoked by childhood illness (*Vocabolario della Crusca*). 

*Caduco* derives from the Latin *cadere* (to fall), hence the English “falling sickness”; the term *caducus* is attested in Apuleius (c. 124–after 170 CE) [2]. Francesco Redi (1626–1697), physician to the Medici court, glossed the vernacular term directly: “*Costui ‘pativa di malcaduco’*,* il qual malcaduco. egli è lo stesso che l’epilessia*” [*this man “suffered from malcaduco*,*” which malcaduco. is the same thing as epilepsy*] (cited in *Vocabolario della Crusca*). 

Independently of its clinical use, *caduco* in early modern Italian carried a second register: transitory, not destined to last, commonly used of worldly things as opposed to the eternal (*Grande Dizionario della Lingua Italiana*, *sub voce* [s.v.] “*caduco*”). The patient falls, and returns. 

Although writing much earlier, Celsus (c. 25 BCE–c. 50 CE) used a different term, *morbus comitialis*, to describe the same transient course in *De Medicina* [3]: the patient suddenly falls, foams at the mouth, then “*after an interval returns to himself*,* and actually gets up by himself.*” He distinguished this course from *lethargus*, a condition marked by languor or torpor and “*an almost insurmountable need of sleep*” in which the patient may not rouse spontaneously. 

The point is not that the word itself contains a theory of consciousness, but that the convulsive phenomenon later designated by terms such as *male caduco* had long been described in terms of reversible loss and recovery of responsiveness.

## Archival sources

Anna Maria Luisa’s recipes should be read within the broader Medici tradition of pharmaceutical and alchemical practice, in which distilled preparations, recipe exchange, and secret remedies linked medical practice, empirical knowledge, and dynastic prestige [1]. Among Anna Maria Luisa’s over 200 recipes, preserved in the *Archivio di Stato di Firenze* (ASF, *Miscellanea Medicea* [MM] 1, ins. 2), two are of particular neurological interest: the first is *Polvere da Infantiglioli* against *male caduco* (ASF, MM 1, ins. 2, fol. 177r); its ingredients and full text are given in the Supplementary Material. 

The second is a preparation of amber essence (ASF, MM 1, fol. 214r), which lists its properties as follows: *“Fortifica il Cervello*,* et il Cuore; Assottiglia l’intelletto; Vivifica i sentimenti; Restituisce la memoria; Rallegra i Melancolie… Giova il suo Profumo allo spasimo*,* alla Paralisia*,* e Mal Caduco”* — strengthening brain and heart, sharpening intellect, animating sensation, restoring memory, alleviating melancholy, treating spasms, paralysis, and the falling sickness.

## Interpretation

The ingredients of the *Polvere da Infantiglioli* — precipitated human skull, oriental pearls, red and white coral, amber, peony root and seeds — merit attention.

The skull, specified as that of a man who died violently and was unburied, reflects a coherent pharmacological logic: seventeenth-century prescriptions across Europe specified precisely this, bone from a man who had met a violent death and remained unburied, as an ingredient against epilepsy, on the belief that violent death left nervous vitality, the *spiritus animalis*, trapped in the bone, available for transfer to a failing brain [4, 5].

Amber draws on an adjacent tradition: its pharmacological use, including preparations for epilepsy, expanded through the post-Paracelsian rise of iatrochemistry, with salts, oils, and tinctures of amber produced by distillation from the early seventeenth century onward [6].

Peony, long used in remedies for epilepsy [2], also appeared in amuletic form: peony-root necklaces were recommended for children’s convulsions in early modern medical texts [7].

These ingredients belonged to the broader early modern *materia medica* for epilepsy. Willis (1621–1675), in *De Anima Brutorum* (1672) [8], rejected a strictly pineal localization of the sensitive soul and placed the *anima sensitiva* within the brain, describing epilepsy as a paroxysmal disturbance capable of interrupting the sensory, responsive, and aware dimension of the living person. The juxtaposition is historically revealing: empirical remedies and emerging neuroanatomical explanations could still converge on the same clinical problem, the sudden interruption of responsiveness, even when they did not yet share a common conceptual language.

Modern pharmacology offers suggestive, but necessarily partial, points of contact: extracts from *Paeonia* roots contain paeoniflorin with anticonvulsant activity [9], and a seizure model in mice found amber to produce metabolic changes comparable to phenobarbital [10]. Such findings suggest that some ingredients belonged to pharmacological traditions that were not merely symbolic. The *male caduco* was being treated with substances embedded in a long empirical pharmacological tradition, before any mechanistic account could explain why seizures impair awareness or how responsiveness returns.

Seen through the lens of modern consciousness science, the two recipes reveal an undifferentiated therapeutic practice directed at functions that modern terminology distinguishes as the level of consciousness, the overall state of arousal and behavioural responsiveness, and its content: what a person perceives, remembers, or feels [11]. Reading the two recipes through this modern nosological framework allows a dialogue between historical practice and contemporary distinctions, clarifying what they do and do not engage with.

The *Polvere da Infantiglioli* concerns episodic convulsive events associated, in modern terms, with transient impairment of the level of consciousness, loss of responsiveness and bodily control, and subsequent recovery. In contemporary epileptology, comparable seizure-related phenotypes are recognized as clinically meaningful alterations of consciousness, distinct in their transient course from the sustained disorders of consciousness such as coma or the vegetative state [11]. In this limited sense, *male caduco* can be discussed retrospectively as one such alteration of consciousness.

The parallel with Celsus remains observational: in *De Medicina*, his descriptions allow a contrast between the seizure, from which the patient “*returns to himself*,” and *lethargus*, a more sustained state from which the patient does not rouse unaided [3]. This contrast does not establish continuity with modern disorders of consciousness, but it does point to a long-standing clinical problem: distinguishing impairments of arousal, awareness, and behavioural responsiveness along a clinical spectrum. In modern neurology, this spectrum encompasses pathological torpor and sleep-like unresponsiveness, impaired spontaneous motor and verbal initiation, as in akinetic mutism, and prolonged disorders of consciousness after severe brain injury [12]. These conditions illustrate how arousal and awareness may be differently affected or may appear dissociated when behavioural responsiveness is impaired. Read in modern terms, restoration of the level of consciousness may allow conscious content to re-emerge, although this may not become immediately evident at the behavioural level.

If the *Polvere da Infantiglioli* can be read primarily in relation to the level of consciousness, the amber essence addresses several functions that modern terminology would predominantly place on the side of conscious content. It is said to sharpen the intellect, vivify sensation, restore memory, and relieve melancholy; yet the same preparation is also prescribed for *male caduco*, without distinction. The claim carries no theoretical apparatus, no account of why one substance should act on all these domains. It suggests instead an implicit therapeutic logic in which functions that contemporary consciousness science would distinguish within conscious content could be addressed together through a single restorative preparation.

Within the broader category of epilepsy and convulsive illness, Anna Maria Luisa’s recipe collection reflects, in part, an eighteenth-century tendency to distinguish infantile convulsive episodes from adult epilepsy [1]. As Buchanan notes, infant convulsions were often viewed as distinct from epilepsy, while adult epilepsy retained an “*air of incurability*” [1]. This tendency was not, however, applied consistently. Alongside the *Polvere da Infantiglioli*, directed specifically at infantile *male caduco*, a third recipe in the same collection, the *Polvere di Fonderia di S. A. Reale* [Powder of the grand-ducal alchemical laboratory (*fonderia*) of His Royal Highness] (ASF, MM 1, ins. 2, fol. 176r) prescribes a single preparation for nursing infants (*fanciulli lattanti*) and adults alike, and under the heading of epilepsy. As a *fonderia* secret, it names no ingredients and specifies only its use, which is why its composition is not discussed here. Where the distinction does appear, it follows age, clinical course, and therapeutic expectation, rather than causal or etiological categories in the modern sense — a reminder that early modern therapeutic practice neither treated all seizure-like episodes as equivalent nor consistently separated them.

## Conclusions

The formal study of disorders of consciousness belongs to the twentieth century; its underlying clinical observation is far older. When a Florentine princess distributed her *Polvere da Infantiglioli* across European courts, she was participating, in her own idiom, in a tradition of observing and attempting to reverse transient losses of responsiveness, a problem that today lies at the border between epileptology and consciousness science.

That this knowledge was held and transmitted by a woman operating outside institutional medicine, through the alchemical laboratory at Villa La Quiete and the epistolary networks of the European nobility, is itself historically significant. Anna Maria Luisa distributed her *Polvere da Infantiglioli* to allied courts across Europe as one of several instruments through which she maintained political relevance during the dissolution of her dynasty [1].

Her case suggests that aristocratic women may have played a largely unacknowledged role in observing and treating convulsive illness, testing, preserving, and transmitting empirical knowledge related to episodes involving transient loss of consciousness long before neurology possessed a formal language for such phenomena.

## Supplementary information

Below is the link to the electronic supplementary material.

Supplementary File 1 (DOCX 14.0 KB)

## Footnotes

1 The Baroness can plausibly be identified as Maximiliana Gräfin Trauttmansdorff, wife of Philipp Ludwig Freiherr von Moltke; their marriage contract is recorded in the Austrian State Archives (Österreichisches Staatsarchiv). Moltke is also documented as having been sent in June 1730 to the Tuscan court of Gian Gastone de’ Medici (1671–1737), Anna Maria Luisa’s brother and the last Medici Grand Duke of Tuscany, providing a plausible link between the Moltke household and the Medici court before the 1735 correspondence discussed here.

2 The last surviving member of the Medici line, Anna Maria Luisa de’ Medici was Electress Palatine by marriage and returned to Florence after the death of her husband, Johann Wilhelm II (1658–1716), in 1716. In 1737 she negotiated the Family Pact (Patto di Famiglia) with the incoming Habsburg-Lorraine dynasty, ate, for the utility of the Public, and to attract the curiosity of Foreigners” (ASF, Trattati Internazionali, 56, ins. 6). The pact proved decisive for preserving Florence’s cultural identity and what would become a central part of Italy’s historical patrimony.
