# Supplementary material for: From male caduco to altered consciousness: two Medici recipes through the lens of modern consciousness science
Source: Neurol Sci. 2026 Aug 7;47(9):689. doi: 10.1007/s10072-026-09309-5 (PMC13451380; doi:10.1007/s10072-026-09309-5)
Supplement: Supplementary file 1 [file 10072_2026_9309_MOESM1_ESM.docx]

### **Supplementary Material**

**Original recipe texts, with English translation**

**S1. Polvere da Infantiglioli contro il Male Caduco o sia mitrito** Archivio di Stato di Firenze (ASF), Miscellanea Medicea 1, ins. 2, fol. 177r

**Italian:**

*Ricetta per fare la Polvere da Infantiglioli contro il Male Caduco, o sia mitrito.*

*Sale, o Magistero di Cranio umano di Uomo morto violentemente, e che non sia stato sepolto oncia iii; Sale, o Magistero di Perle Orientali oncia ii; Magistero di Coralli rossi oncia ii; Magistero di Coralli bianchi oncia ii; Sale di Carabe once i; Radice di Peonia once i; Semi di Peonia mondati dal suo guscio once i. Il tutto si polverizzi, e si passi per staccio fine, e si unisca bene insieme, e di detta Polvere se ne dà Grani cinque per presa.*

**English translation:**

Recipe for making the Infantiglioli Powder against the Falling Sickness, or mitrito.

Salt, or Magistery, of human Skull of a Man who died violently, and who was not buried, 3 ounces; Salt, or Magistery, of Oriental Pearls, 2 ounces; Magistery of red Coral, 2 ounces; Magistery of white Coral, 2 ounces; Salt of Carabe, 1 ounce; Peony Root, 1 ounce; Peony Seeds, cleaned of their shell, 1 ounce. Let everything be pulverized, and passed through a fine sieve, and mixed well together, and of said Powder five Grains are given per dose.

**S2. Essenza d'Ambra** Archivio di Stato di Firenze (ASF), Miscellanea Medicea 1, fol. 214r

**Italian:**

*L'Essenza d'Ambra Fortifica il Cervello, et il Cuore; Conforta i membri deboli; Assottiglia l'intelletto; Vivifica i sentimenti; Restituisce la memoria; Rallegra i Melancolie; Disoppila la matrice; Giova il suo Profumo allo spasimo, alla Paralisia, e Mal Caduco; Correggie l'aere Pestifere, et è un eccellentissimo corroborativo a i Vecchi et a chi è frigido di Complessione; Se ne danno 12 o 16 goccie in qualche liquore appropriato.*

**English translation:**

The Essence of Amber Fortifies the Brain and the Heart; Comforts the weak members; Sharpens the intellect; Vivifies the senses; Restores the memory; Gladdens the Melancholies; Unblocks the womb; its Scent benefits spasm, Paralysis, and the Falling Sickness; Corrects pestilential air, and is a most excellent restorative for the Elderly and for whoever is of cold Complexion; 12 or 16 drops are given in some appropriate liquor.
